# Advanced Mathematical Study and the Development of Conditional Reasoning Skills

**DOI:** 10.1371/journal.pone.0069399

**Published:** 2013-07-15

**Authors:** Nina Attridge, Matthew Inglis

**Affiliations:** 1 Centre for Pain Research, University of Bath, Bath, United Kingdom; 2 Mathematics Education Centre, Loughborough University, Loughborough, United Kingdom; University of Minnesota, United States of America

## Abstract

Since the time of Plato, philosophers and educational policy-makers have assumed that the study of mathematics improves one's general ‘thinking skills’. Today, this argument, known as the ‘Theory of Formal Discipline’ is used in policy debates to prioritize mathematics in school curricula. But there is no strong research evidence which justifies it. We tested the Theory of Formal Discipline by tracking the development of conditional reasoning behavior in students studying post-compulsory mathematics compared to post-compulsory English literature. In line with the Theory of Formal Discipline, the mathematics students did develop their conditional reasoning to a greater extent than the literature students, despite them having received no explicit tuition in conditional logic. However, this development appeared to be towards the so-called defective conditional understanding, rather than the logically normative material conditional understanding. We conclude by arguing that Plato may have been correct to claim that studying advanced mathematics is associated with the development of logical reasoning skills, but that the nature of this development may be more complex than previously thought.

## Introduction

“Those who have a natural talent for calculation are generally quick at every other kind of knowledge; and even the dull, if they have had an arithmetical training […] become much quicker than they would otherwise have been.” (Plato [1] p. 256)

For millennia it has been assumed that people can be taught to think more logically, and in particular, that mathematics is a useful tool for doing so. This idea is known as the Theory of Formal Discipline (TFD) and dates from the time of Plato. It is exemplified by the philosopher John Locke's suggestion that mathematics ought to be taught to “all those who have time and opportunity, not so much to make them mathematicians as to make them reasonable creatures” [2] Similarly, the contemporary mathematician Amitsur argued that “through mathematics we also wish to teach logical thinking – no better tool for that has been found so far” [3].

In view of its intellectual pedigree and clear policy implications, variants of the TFD are regularly cited in educational policy debates and curricula reform documents [4], [5]. The National Council for Teachers of Mathematics' (NCTM) Principles and Standards, for example, stated that studying mathematics is important because “students who can use many types of reasoning and forms of argument will have resources for more effective reasoning in everyday situations” ([6], p.345). Similarly, in a report to the UK government, Smith [7] argued that mathematics education “disciplines the mind, develops logical and critical reasoning, and develops analytical and problem-solving skills to a high degree” (p.11).

Society's views on the TFD have important practical implications. Stanic [8] noted that changes to the US school-level mathematics curriculum have been substantially related to views about the veracity of the TFD. The theory also appears to be implicitly endorsed by the employment market: in the UK, workers who have studied post-compulsory mathematics earn, at the age of 33, 7–10% more than those with similar ability and qualifications [9]. Clearly the study of advanced mathematics is valued by employers and policy-makers. Although this is largely because mathematical knowledge is important in its own right, it also appears to be influenced by the belief that studying mathematics makes one more ‘logical’. The question that naturally arises concerns whether the TFD is accurate: does studying advanced mathematics develop one's logical reasoning skills?

### Training reasoning skills

Psychological evidence relating to the TFD is inconclusive. Thorndike [10] measured the effect of one year of schooling in various combinations of subjects on performance on an intelligence test. His findings revealed small improvements associated with the study of French, chemistry and trigonometry, while arithmetic, geometry and algebra were associated with improvements barely above zero. These and other findings (e.g., [11], [12]) have led many researchers to conclude that reasoning skills cannot be divorced from the context in which they are learnt, and therefore to reject the TFD.

Cheng, Holyoak, Nisbett and Oliver [13] found that even training in formal logic did not improve performance on a conditional logic task. Their participants were given 40 hours of training in the logic of the conditional, including modus ponens, modus tollens, denial of the antecedent, affirmation of the consequent and the distinction between the conditional and biconditional. Despite such comprehensive training, they found no significant improvement in performance on four Wason selection tasks [14]. However, this result is not easy to interpret given the varying contexts in which Cheng et al. situated their problems. More recently researchers have questioned whether selection tasks, and in particular contextualized selection tasks, measure conditional reasoning at all (e.g., [15], [16]).

Despite these negative findings, there has been some support for the idea that studying mathematics might develop conditional reasoning ability. Lehman and Nisbett [17] tracked the development of statistical reasoning, verbal reasoning and conditional reasoning in US undergraduates over their four years of study. Although they did not study mathematics students, they did find a significant correlation between improvement in conditional reasoning and the number of mathematics courses taken by the natural science students in their sample. However, their conditional reasoning test consisted of only one abstract, one causal-framed and one permission-framed Selection Task, and one biconditional Selection Task, and so suffered from the same limitations as that used by Cheng et al. [13].

Inglis and Simpson [18] found that mathematics undergraduates ‘outperformed’ intelligence-matched comparison undergraduates on a 32-item abstract conditional inference task [19]: in other words that their behavior more closely matched a material interpretation of conditionals (discussed below). However, across the course of their first year of studies there were no changes in reasoning behavior. Inglis and Simpson offered two possible explanations for the initial between-groups difference on entry to university: either those who are more likely to adopt the material conditional are disproportionately filtered into studying university-level mathematics, or that studying post-compulsory but pre-university mathematics influences conditional reasoning behaviour. This latter account is plausible because in England (where, like ours, Inglis & Simpson's study was conducted) students are able to drop mathematical study at age 16. A minority choose to study it at ‘Advanced Level’ (commonly referred to as A-Level), a two year course, the results of which are used by universities to select incoming undergraduates. Students typically take three or four subjects at A-Level, of which mathematics might be one, or (rarely) two. It might be that studying A-Level mathematics develops one's ability to reason logically, although the A-Level syllabus contains no tuition on conditional statements. A third possibility is that the difference found by Inglis and Simpson was due to between-group differences unrelated to intelligence (which they controlled for), such as thinking dispositions. In this paper we aim to distinguish between these three hypotheses.

### Models of the Conditional

Abstract conditional reasoning consists of drawing conclusions from a conditional statement ‘if *p* then *q*’ and a premise. Here we restrict our interest to what Evans, Handley, Neilens & Over [20] referred to as basic conditionals: those concerning abstract relationships which are, at least in principle, empirically verifiable (e.g. “if there is a T on the card, then there is a 7 on the card”). Four inferences are typically drawn by participants: modus ponens (MP), denial of the antecedent (DA), affirmation of the consequent (AC) and modus tollens (MT). These inferences are summarised in Table 1 (so an example of a DA inference would be to conclude not-7 from the premises ‘if T then 7’ and not-T). The MP, DA, AC and MT inferences are respectively drawn by around 100%, 55%, 75% and 60% of reasoners [20].

**Table 1:** The four inferences (modus ponens, denial of the antecedent, affirmation of the consequent and modus tollens) with and without negated premises (Prem) and conclusions (Con).

	MP		DA		AC		MT	
	Prem	Con	Prem	Con	Prem	Con	Prem	Con
if *p* then *q*	*p*	*q*	not-*p*	not-*q*	*q*	*p*	not-*q*	not-*p*
if *p* then not-*q*	*p*	not-*q*	not-*p*	*q*	not-*q*	*p*	*q*	not-*p*
if not-*p* then *q*	not-*p*	*q*	*p*	not-*q*	*q*	not-*p*	not-*q*	*p*
if not-*p* then not-*q*	not-*p*	not-*q*	*p*	*q*	not-*q*	not-*p*	*q*	*p*

Validity				
Material Conditional	Valid	Invalid	Invalid	Valid
Defective Conditional	Valid	Invalid	Invalid	Invalid
Biconditional	Valid	Valid	Valid	Valid
Conjunction	Valid	Invalid	Valid	Invalid

The validity of each inference is shown for the material, defective, biconditional and conjunction interpretations of the conditional.

The validity of these four inferences depends upon how the reasoner interprets ‘if *p* then *q*’. Here we briefly summarise four possible interpretations of basic conditionals: the material conditional, the biconditional, the defective conditional, and the conjunctive conditional. Truth tables for these interpretations are shown in Table 2.

**Table 2:** Truth tables for the material, defective, biconditional and conjunction interpretations of the conditional (T – true; F – false; I – irrelevant).

p	q	if p then q			
		Material	Defective	Biconditional	Conjunction
T	T	T	T	T	T
T	F	F	F	F	F
F	T	T	I	F	F
F	F	T	I	T	F

The material conditional ‘if *p* then *q*’ is true except when *p* and not-*q* are true; under this interpretation the MP and MT inferences are valid, and the DA and AC inferences invalid. Although the material interpretation is that favored by logicians, it is clear that this is not the meaning which arises in day-to-day conversation, as it has the paradoxical consequence that the truth of *not-*p implies anything (“if Maastrict is in Belgium, then Rome is in Italy” is a true statement under the material conditional). While proponents of the TFD typically fail to explicitly state which model of the conditional they believe mathematical study promotes, we interpret claims about the development of “man's purely logical faculties” ([21], p. 19) as most likely being concerned with the (logically normative) material conditional.

Under the biconditional interpretation all four inferences are valid: ‘if *p* then *q*’ is interpreted to mean ‘*p* if and only if *q*’. Although this could be a conjunction of two material conditionals, Evans et al. [20] suggested that at least some reasoners who adopt the biconditional are actually using a ‘simple equivalence’ strategy. Rather than conjoining two materials, they merely expect that *p* and *q* must go together (hence MP and AC), and that not-*p* and not-*q* must go together (hence DA and MT).

Some reasoners believe that ‘if *p* then *q*’ is only relevant when *p* is true [22]. Under this so-called ‘defective’ interpretation only MP is (immediately) valid. DA, AC and MT are not since none involves a *p* premise, so the conditional adds no additional information. Nevertheless, it is possible to draw the MT inference under a defective interpretation using a complex combination of MP and a contradiction argument (assume for contradiction *p*, conclude *q* by MP, but this is a contradiction with the minor premise not-*q*, so the assumption *p* cannot be correct, hence not-*p*). Mental logic theorists suggest that the relative complexity of this string of deductions is why MT is not as frequently made as MP (e.g., [23]). Finally, reasoners may interpret ‘if *p* then *q*’ to mean simply ‘*p* and *q*’ [24]. Under this conjunctive interpretation both MP and AC are valid, but neither DA nor MT are (since neither has a *p* or *q* premise).

Theories of reasoning differ on the causes of the different interpretations. For example, the mental models theory [25] suggests that reasoners typically represent a conditional ‘if *p* then *q*’ with one explicit mental model, together with an implicit model that denotes the possible existence of not-*p* cases:
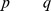



Some high-ability reasoners may flesh out the implicit model (a cognitively demanding task), giving them access to the material conditional and the MT inference. But reasoners who forget about the implicit model, or who lack the working memory capacity to flesh it out, are left with their initial explicit model, leading to either the defective or conjunctive interpretation.

In contrast, Evans et al.'s [20] suppositional account suggests that there are two groups of reasoners: (i) a less sophisticated group who see the probability of a conditional P(if *p* then *q*) as being equal to the probability of the conjunction P(*p* & *q*), this results in the ‘simple equivalence’ strategy discussed earlier; and (ii) a more sophisticated group who see it as being equal to the conditional probability P(*q*|*p*), which results in the defective interpretation. Evans et al. suggested that their account can be distinguished from the mental models theory by considering the MT inference. Under Johnson-Laird & Byrne's [25] account, the MT inference should be made by relatively high-ability reasoners (since it involves the fleshing out of implicit models). In contrast the suppositional account suggests that higher-ability participants should draw the MT inference *less*, as it does not immediately follow from the defective interpretation. In support of this latter account, several studies have found that measures of intelligence are negatively correlated with the frequency of the MT inference [20], [26].

Our goal here was to determine whether, as predicted by TFD, studying mathematics impacts upon students' conditional reasoning. In particular, we investigated whether the extent to which students adopted the material, biconditional, defective and conjunctive interpretations of the conditional changed following a year of mathematical study.

While the TFD claims that studying mathematics develops one's reasoning skills, it does not suggest any cognitive mechanisms for the change. Reasoning performance is related to measures of cognitive capacity (i.e., general intelligence; [20], [26], [27]) and thinking dispositions (i.e., the tendency to use one's cognitive capacity to solve problems; [28]). It is therefore possible that if studying mathematics did change conditional reasoning behavior it might do so via changes in either cognitive capacity or thinking disposition. Here we investigated whether either of these possibilities could provide plausible mechanisms by which the TFD might operate.

Of course, it is neither practical nor ethical to randomly assign participants to courses when high-stakes qualifications are at stake. However, our inclusion of a comparison group who were studying English literature allowed us to attenuate the non-random assignment to conditions to some extent. The comparison group allowed us to distinguish changes that occur simply due to age or education from those specifically related to some aspect of (or related to a factor correlated with) studying mathematics.

### Summary

In sum, we asked two main questions. First, does studying post-compulsory mathematics influence how one reasons with conditionals? Second, if there is development of conditional reasoning skills, is this the result of a domain-general change in cognitive capacity or thinking disposition?

## Method

### Participants

One hundred and twenty four participants (aged 15 years 4 months–17 years 8 months, M = 16 years 6 months, at Time 1) were recruited from five schools in Leicestershire, Hampshire and Derbyshire, UK. Seventy-seven (41 male) were studying mathematics amongst any other subjects and 47 (17 male) were studying English literature and not mathematics. The literature students served as a comparison group. To avoid factors such as stereotype threat [29] influencing responses, participants were not told about the specific hypothesis, or about the mathematics versus literature comparison. All participants provided written informed consent, and the study was approved by Loughborough University's Ethical Advisory Committee.

### Design

The study followed a longitudinal quasi-experimental design. Participants were recruited after they had chosen their post-compulsory subjects and were tested at the beginning (during the first term and as close to the start of term as possible) and end (after teaching had finished) of their first year of post-compulsory study. They completed the same set of tasks at both time points.

### The Mathematics Syllabus

Participants in the mathematics group were all studying the first year of Advanced-Level mathematics. Although there are three different versions of this course available to students in England, all have similar content. Among other topics, the syllabus contained sections on algebra, geometry, calculus, trigonometry, probability, mathematical modeling, kinematics and forces (e.g., [30]). Most importantly, students were not taught any proof-based mathematics, nor were they taught the definition of the conditional statement. To formally establish this, as well as inspecting the syllabus, we conducted an analysis of every first year A-level mathematics examination between 2009 and 2011. Of 929 questions set, only one contained an explicit “if…then” sentence, and there were no mentions at all of the terms “modus ponens”, “modus tollens” or “conditional”.

### Measures

#### Conditional Inference

**Figure 1:**
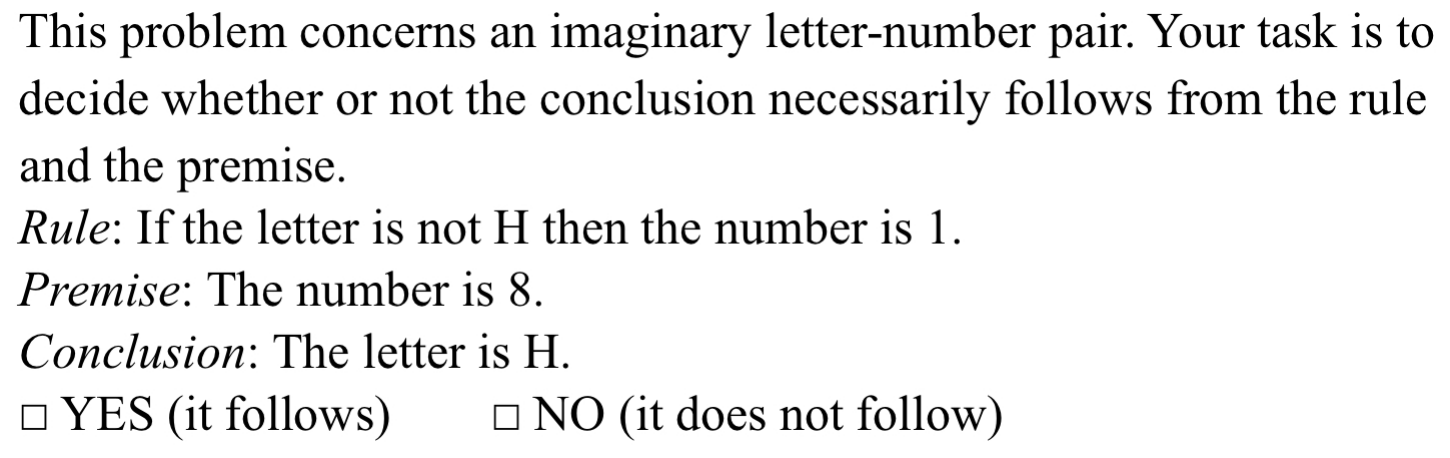
Four indicative items from the Conditional Inference Task (adapted from [19]). The problems ask about a) the *modus ponens* inference, b) the *denial of the antecedent* inference, c) the *affirmation of the consequent* inference, and d) the *modus tollens* inference.

Participants completed Evans et al.'s [19] version of the Conditional Inference Task. The task consists of 32 abstract items of four inference types: MP, DA, AC and MT. The inferences used are shown in Table 1; half of the problems used explicitly negated premises (e.g. not-4 was represented as “not 4”) and half used implicitly negated premises (e.g. not-4 was represented as, for example, “8”). The lexical content of the rules were generated randomly and the order of the problems was randomized for each participant. The instrument was preceded by the instructions used by Evans et al. An indicative item of each inference type is shown in Figure 1.

#### Cognitive Capacity: Raven's Advanced Progressive Matrices (RAPM)

An 18 item subset of RAPM with a 15 minute time limit was used as a measure of cognitive capacity [31], [32].

#### Thinking Dispositions: Cognitive Reflection Test (CRT)

**Figure 2:**
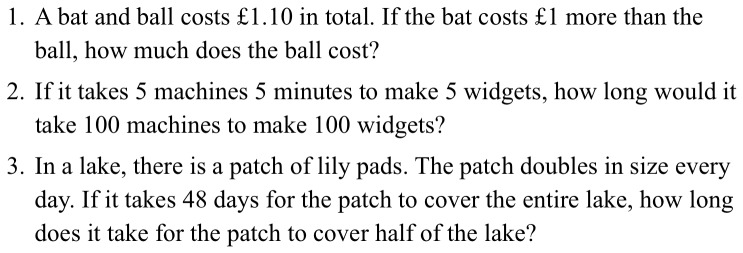
The three items from the Cognitive Reflection Test [33].

As suggested by Toplak et al. [28], we used the number of intuitive responses given to the three-item CRT [33] as a (reverse-scored) performance measure of participants' rational thinking dispositions. Toplak et al found the CRT to be a better predictor of rational responding to reasoning tasks than cognitive ability, executive functions, or the 41-item Actively Openminded Thinking scale. These questions, shown in Figure 2, were randomly intermixed with three simple mathematical word problems of a similar length from the Woodcock-Johnson III Applied Problems subtest. This was intended to prevent the ‘trick’ nature of the CRT questions from being recalled at the second time point. We also included the self-report Need for Cognition Scale [34] as an additional measure of thinking disposition, but found no between-groups differences, nor any development during the course of the year (*p*s>.4), and therefore omit further discussion of these data.

#### Prior Academic Attainment

We asked participants to report their General Certification of Secondary Education (GCSE, the examinations taken by 16 year-old school leavers in England) grades. Each grade was converted to an 8-point scale (A* = 8, A = 7, etc) and summed to produce a total score.

#### Mathematics Manipulation Check

A 15-item mathematics test was included as a manipulation check. Twelve items were taken from the Woodcock-Johnson III Calculation subtest. Nine had shown an average accuracy of less than 55% and correlated with performance on the whole test at.86 in a previous dataset with mixed-discipline undergraduate students [35]. Three items were taken from the lower range to prevent floor effects in the literature group. The final three items on the test were the most difficult items on the Loughborough University diagnostic test for incoming mathematics undergraduates, based on performance in 2008 and 2009. Questions were presented in a set order that was intended to be progressive.

### Procedure

Participants took part in groups (5–34) during the school day under examination conditions. All tasks were given in a single paper booklet. The RAPM task was always completed first with a 15 minute time limit, and the order of the subsequent tasks was counterbalanced between-participants following a Latin Square design. Participants were instructed to work at their own pace until they had completed all tasks and the sessions lasted approximately 45 minutes.

## Results

### Preliminary analyses

#### Data inclusion

Forty-four mathematics students and thirty-eight literature students took part at both time points and were included in the analysis. Those who dropped out of the study had typically moved schools or changed courses; there were no significant differences in Time 1 scores on any of the measures between those who took part at Time 2 and those who dropped out (*p*s>.15).

#### Covariates

Descriptive statistics for the various covariates are shown in Table 3. At Time 1, the mathematics group scored significantly higher on the RAPM, *t*(79) = 3.38, *p* = .001, and CRT, *t*(79) = 4.79, *p*<.001, and had marginally higher prior academic attainment, *t*(122) = 3.89, *p* = .089, than the literature group. Furthermore, the RAPM, *r* = .417, *p*<.001, CRT, *r* = .417, *p*<.001, and prior academic attainment, *r* = .304, *p*<.001, scores were significantly correlated with the extent to which conditional inferences were evaluated in line with the material conditional conception (defined below as the material conditional index). Consequently RAPM, CRT and prior attainment are used as covariates in subsequent analyses. Although both groups improved their RAPM and CRT scores slightly over the course of the year, neither Group × Time interaction effect approached significance, *p*s>.2.

**Table 3:** Means and standard deviations for measures of covariates and mathematical achievement.

		Mathematics		Literature	
	Theoretical maximum	Mean	Std Dev	Mean	Std Dev
Time 1 RAPM	18	9.64	3.32	6.94	3.54
Time 1 CRT intuitive (reverse scored)	3	1.79	1.14	.89	.85
Time 1 Mathematics	15	4.86	1.59	3.50	.97
Prior academic attainment	–	66.45	9.78	61.53	14.03
Time 2 RAPM	18	10.64	2.93	7.32	3.15
Time 2 CRT intuitive (reverse scored)	3	1.98	1.02	1.11	1.02
Time 2 Mathematics	15	6.95	1.94	3.19	.57

Units are number of correct responses except for prior academic attainment, which is sum of grades for all GCSEs where A* = 8, A = 7, B = 6 etc.

#### Manipulation Check

The mathematics group showed significantly greater improvement on the mathematics test than the literature students, *F*(1,79) = 46.324, *p*<.001, confirming that as a group they engaged with and learned from their year of studying mathematics.

### Conditional Inference Scores

**Figure 3:**
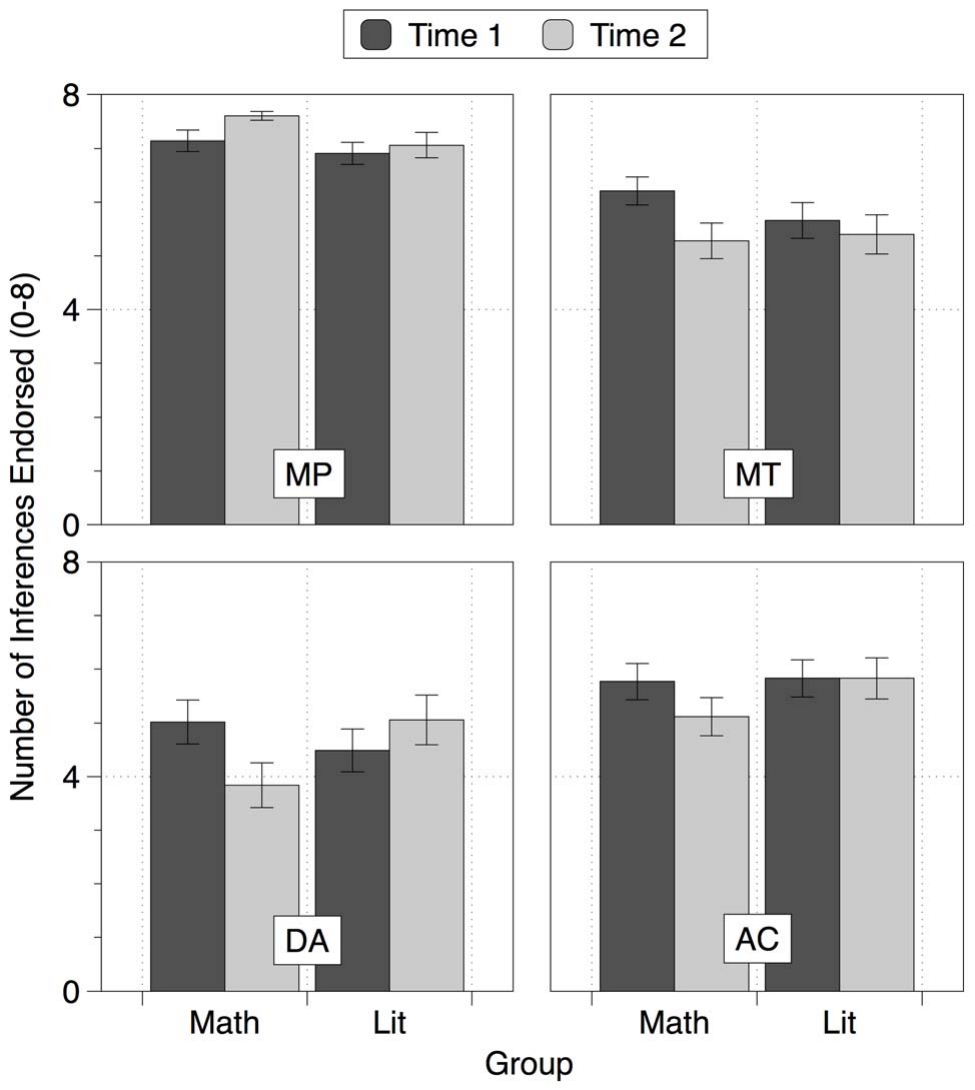
The number of inferences endorsed by each group, for MP, MT, DA and AC inferences at the two time points. Error bars show ±1 SE of the mean.

Endorsement rates of each inference type were analysed with a 2×4×2 ANOVA with two within-subjects factors: Time (start and end of the year) and Inference Type (MP, DA, AC, MT), and one between-subjects factor: Group (mathematics and literature). This revealed a significant three-way interaction, *F*(3,228) = 7.476, *p*<.001, *η_ρ_^2^* = .090, shown in Figure 3, which remained significant after controlling for Time 1 RAPM, Time 1 CRT and prior academic attainment, *F*(3,216) = 5.103, *p* = .002, *η_ρ_^2^* = .066. Compared to Time 1, the mathematics students at Time 2 endorsed more MP inferences, *t*(42) = 2.420, *p* = .020, *d* = .413, and rejected more DA, *t*(42) = 3.978, *p*<.001, *d* = −.607, AC, *t*(42) = 3.060, *p* = .004, *d* = −.468, and MT inferences, *t*(42) = 2.877, *p* = .006, *d* = −.446. In contrast, the literature group showed no significant differences between Time 1 and Time 2 scores for any inference, although there was a marginally significant increase in the number of DA inferences endorsed, *t*(34) = 1.795, *p* = .082, *d* = .309.

**Figure 4:**
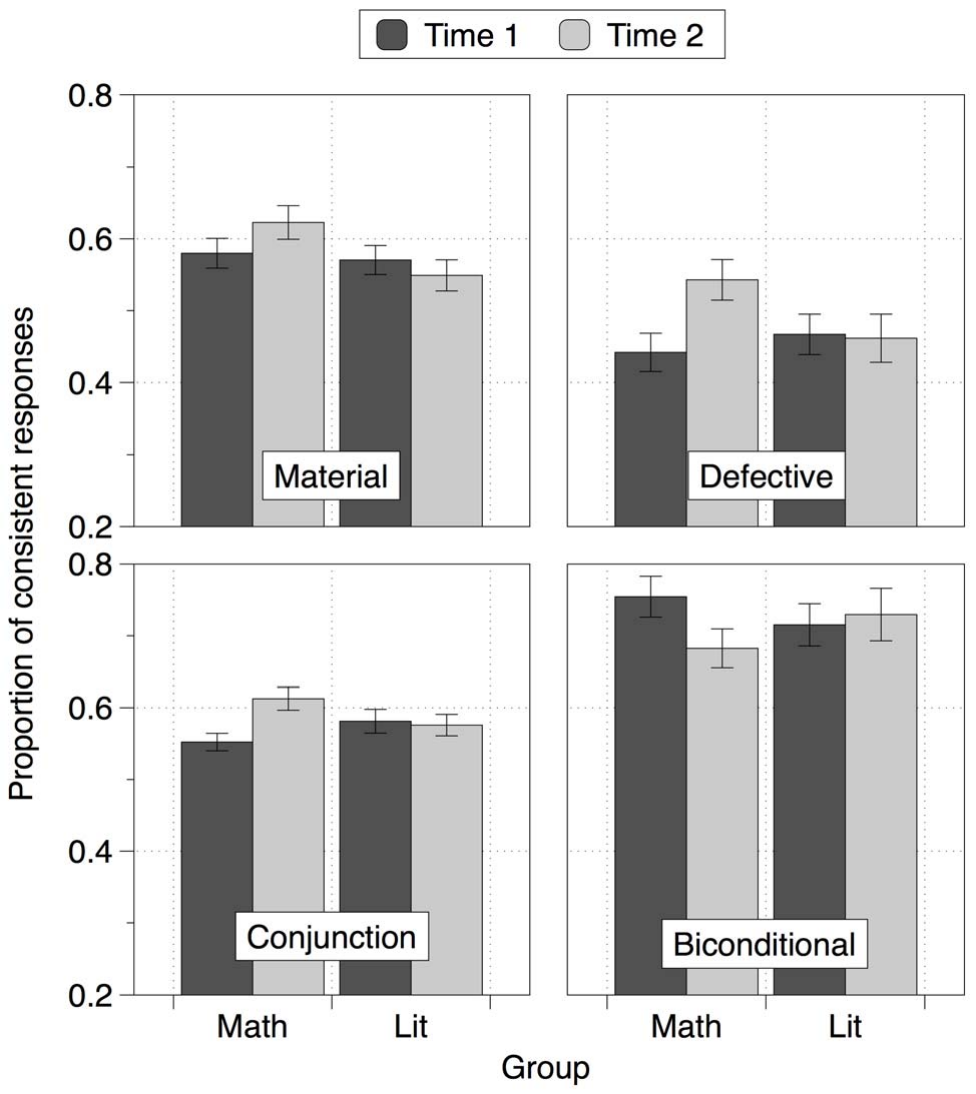
Conditional Inference scores of the two groups at Time 1 and 2, with reference to the four different interpretations of the conditional. Error bars show ±1 SE of the mean.

These responses appear most consistent with an increased tendency for the mathematics students to adopt a defective conditional interpretation (more MP inferences and fewer DA, AC and MT inferences were made at Time 2 compared to Time 1). To test for this we calculated four indices for each participant (at each time point) giving the proportion of responses consistent with each of the four interpretations of the conditional. For example, a person responding entirely in line with the material conditional would respond ‘yes’ to all MP and MT inferences and ‘no’ to all DA and AC inferences. A Material Conditional Index was therefore computed as: number of MP inferences endorsed + number of MT inferences endorsed + (8 – number of DA inferences endorsed) + (8 – number of AC inferences endorsed), giving a material conditional index score out of 32 for each participant at each time point. The consistency scores for each interpretation are shown in Figure 4, and were subjected to a series of 2×2 ANOVAs with one within-subjects factor: Time (start and end of the year) and one between-subjects factor: Group (mathematics, literature). We consider each interpretation in turn.

On the material conditional analysis Group and Time interacted, *F*(1,76) = 11.860, *p* = .001, *η_ρ_^2^* = .135 (*p* = .007 with covariates). The mathematics group became more material, *t*(42) = 3.171, *p* = .003, *d* = .493, whereas the literature group did not change, *p* = .092. On the biconditional analysis, Group and Time also interacted, *F*(1,76) = 7.966, *p* = .006, *η_ρ_^2^* = .095, although this was only marginally significant when covariates were included, *F*(1,72) = 3.697, *p* = .058, *η_ρ_^2^* = .049. The mathematics group became less biconditional, *t*(42) = 3.323, *p* = .002, *d* = −.508, whereas the literature group did not change, *p* = .500.

On the defective analysis, Group and Time interacted, *F*(1,76) = 17.651, *p*<.001, *η_ρ_^2^* = .188 (*p* = .002 with covariates). The mathematics group became more defective, *t*(42) = 5.756, *p*<.001, *d* = .880, whereas the literature group did not change, *p* = .767. Finally, on the conjunctive analysis, Group and Time also interacted, *F*(1,76) = 8.525, *p* = .005, *η_ρ_^2^* = .101 (*p* = .014 with covariates). The mathematics group became more conjunctive, *t*(42) = 3.534, *p* = .001, *d* = .548, whereas the literature group did not change, *p* = .693.

Comparing the effect sizes of these analyses confirms that the change in the mathematics group is best understood as an increased tendency to adopt the defective interpretation of the conditional. In other words, that over time the mathematics group became more likely to endorse the MP inference, but less likely to endorse the DA, AC and MT inferences. Next we considered whether changes in either cognitive capacity or thinking disposition could represent domain-general mechanisms for this change in conditional reasoning behavior.

### Mechanisms of Development

To investigate whether changes in the domain-general reasoning measures could account for the changes in the mathematics group's conditional reasoning behaviour, we regressed participants' defective conditional change scores (Time 2 defective conditional index minus Time 1 defective conditional index) against their Time 1 RAPM and CRT scores, their prior academic attainment, their RAPM and CRT change scores (the difference between their Time 2 and Time 1 scores), the group they were in, and the two group by change-score interaction terms. If the increased defective conditional indices of the mathematics students could be accounted for by changes in domain general factors, we would expect that some of the change scores or the group by change-score interactions would be significant predictors. However, if the primary factor was the experience of studying mathematics, we would expect the group factor to be the only significant predictor.

The regression model is presented in Table 4. The only significant predictor of change in defective conditional scores was Group, *β* = .337. None of the change scores, nor the change by group interactions approached significance. This analysis seems to suggest that the change in conditional reasoning behavior in the mathematics group is most likely to be related to experiences gained in their mathematical study, not to domain-general changes in cognitive capacity or thinking disposition.

**Table 4:** A regression analysis, predicting change in defective conditional index scores.

R^2^	Predictors	B	Std. Error	β
.253**	Initial RAPM	.003	.005	.093
	Initial CRT	.012	.017	.107
	Prior attainment	.000	.001	−.019
	RAPM change	.010	.008	.217
	CRT change	.012	.021	.084
	Group (0 = literature, 1 = mathematics)	.082	.033	.337*
	RAPM change × Group	.004	.010	.067
	CRT change × Group	−.023	−.032	−.106

*p<.05;

**p<.01.

## Discussion

Since Plato asserted that studying mathematics improves one's ‘quickness’ of thought, philosophers, educational policy-makers and the employment market have placed a high value upon having an advanced education in mathematics. Here we asked whether Plato's position is reasonable; in particular, we asked whether studying post-compulsory mathematics is associated with a development in conditional reasoning behavior, even if that study contained no explicit reference to conditional logic. We found that students studying post-compulsory mathematics did change their reasoning behavior to a greater extent than a comparison group over the course of a year of post-compulsory mathematical study. Further, we found that this change appeared to be best described as development away from a biconditional understanding of the conditional, and towards a defective understanding: at the end of their studies, the mathematics group endorsed more MP inferences and fewer DA, AC and MT inferences. Finally, we demonstrated that this effect was not the result of a domain-general change in cognitive capacity or thinking disposition, but rather seems most likely to be associated with the domain-specific study of mathematics.

Inglis and Simpson [18] found that, compared to intelligence-matched comparison undergraduates, incoming mathematics undergraduates reasoned differently on the conditional inference task used here, but that they did not change over a year of mathematical study. The authors suggested that the initial difference may have been due to one of three possibilities which we aimed to distinguish between: post-compulsory but pre-university study of mathematics developing reasoning skills; filtering of more material reasoners into the study of mathematics; or between-group differences unrelated to intelligence, such as in thinking disposition. Our findings are consistent with the first possibility, that the post-compulsory pre-university study of mathematics develops conditional reasoning skills. At the start of post-compulsory education, the students studying mathematics in our sample did not differ from non-mathematics students on the conditional reasoning task, but they did after a year of study. This change was not due to between-group differences in initial or changed thinking disposition (or cognitive capacity). However, the change was best characterized as a move away from the biconditional interpretation of the conditional towards the defective interpretation, not towards the material interpretation favored by logicians.

Given that Cheng et al. [13] found no change in conditional reasoning scores after a semester of studying formal logic, it may seem surprising that we found that studying mathematics (with no formal logic component) *was* associated with a development in conditional reasoning. We see two ways of accounting for this apparent discrepancy. First, as discussed earlier, Cheng et al. [13] used a series of variants of the Wason Selection Task [14] as their dependent measure. It may be that, as Sperber et al. [15] have argued, the Selection Task is simply not a measure of conditional reasoning ability. Perhaps if Cheng et al. had used a task that was more straightforwardly related to conditional inference they would have found an effect.

An alternative possibility is that the study of mathematics influences conditional reasoning behavior in a different way to the study of formal logic and that this, in some cases at least, is more educationally effective. This possibility is plausible for two reasons. First, we found that development in conditional inference was not related to changes in intelligence or thinking disposition, suggesting that studying mathematics could provide some specific experiences of manipulating concepts logically, which may not be provided by studying logic (we speculate below on what these experiences could be). Second, we found that the mathematics students in our sample did not become consistently more material across inference types, as we would expect if they had simply developed a more normative understanding of conditional statements (which presumably would be the aim of an education in formal logic). In fact, a defective interpretation is unlikely to lead to normative responses to the Wason Selection Tasks used by Cheng et al. (one might expect that reasoners adopting such an interpretation would choose the true antecedent card and no others, rather than selecting the normatively correct true antecedent and false consequent cards).

What then could be the nature of the experiences provided by mathematical study that could develop a defective interpretation of the conditional? Mathematics as a discipline is concerned with deducing the consequences of assumptions. Even before a student begins to study advanced-level mathematical proofs and axiomatic systems, their day-to-day activity consists of making modus ponens deductions from assumptions. Consider, for example, the activity of solving an equation. One starts with an assumption, *f*(*x*) = *x* ^2^+8*x*+19 = 0 say, and is required to determine what follows. For example, a student might deduce that (*x*+4)^2^+3 = 0, and conclude that *f*(*x*) has a minimum at 3, and therefore that *f*(*x*) = 0 has no real solutions. It is notable that the logical manipulations required here are all forward in direction: they require the student to assume that *p* is true and deduce some appropriate *q*. This line of reasoning is incompatible with a biconditional reading of the conditional (taking such a interpretation would require one to believe that *f*(*x*) = 0 having no real solutions is equivalent to *f*(*x*) having a minimum at 3). It is not until students are introduced to proofs by contradiction that they are regularly required to make modus tollens deductions; and students are known to find the transition to indirect proving extremely challenging (e.g. [36], [37]).

Our findings also have implications for the debate between those who favor the suppositional account of conditional reasoning (e.g., [20]) and mental models theorists (e.g. [25]). Recall that the mental models theory attributes reasoners' failure to make the MT deduction to their unwillingness or inability to ‘flesh out’ the implicit mental model contained alongside their initial *pq* model. Thus mental models theorists would predict that reasoners of higher ability would be more likely to make the MT deduction. In contrast the suppositional account suggests that reasoners of high ability are less likely to make the MT deduction, as they are likely to adopt a supposition P(*q*|*p*) model of the conditional rather than the more limited conjunction P(*p*&*q*) model. Evans et al. found empirical support for the latter position; that those participants with higher scores on an intelligence test were less likely to draw the MT deduction. Our data can be seen as a stronger within-subjects test of the suppositional account. We found that studying mathematics was associated, within subjects, with a reduced likelihood to draw the MT deduction, and increased adoptance of the defective interpretation. It seems extremely hard to reconcile this finding with the mental models account. To do so would require that the individual participants lost the ability or willingness to flesh out their implicit model as a consequence of studying advanced mathematics.

Finally, it is important to consider the limitation that results from the quasi-experimental design of our study: we cannot infer that if all students were compelled to study advanced mathematics there would be a society-wide change in conditional reasoning behavior. It remains a possibility that the TFD only applies to those who have *chosen* to study advanced mathematics. Perhaps it requires a certain keenness to learn and to engage with the course material in order for a student to develop in the fashion that we have observed. It is therefore possible that where is it compulsory to study mathematics until 18, as is the case in most non-UK contexts [38] not all students will develop their conditional reasoning skills in the fashion we observed here. Indeed, cross-cultural comparisons of the development of logic skills in students studying different curricula (and in particular curricula where studying mathematics is and is not compulsory until the age of 18) would be a useful direction for future research.

To summarize, our study has provided evidence that the claims made by Plato [1] and John Locke [2] highlighted at the start of the paper have some merit: contrary to Thorndike's [10] early findings, studying mathematics at advanced levels is associated with development of logical reasoning skills.
